# Development and evaluation of an integrated clinical nutrition management system for inpatients

**DOI:** 10.3389/fnut.2026.1971974

**Published:** 2026-10-27

**Authors:** Haili Shan, Quan Zhou, Wei Hu, Yi He, Huifang Jiang, Wei He, Haibin Dai

**Affiliations:** Department of Pharmacy, The Second Affiliated Hospital, Zhejiang University School of Medicine, Hangzhou, China

**Keywords:** clinical decision support systems, clinical nutrition, digital medicine, enteral nutrition, healthcare information system, individualized nutritional therapy, parenteral nutrition

## Abstract

**Background:**

Malnutrition is highly prevalent in hospitalized patients. Standardized nutritional interventions have been associated with improved clinical outcomes and reduced healthcare expenditures. Fragmented workflows, challenges in interdisciplinary coordination, and the absence of structured digital decision support often compromise the quality of nutritional care for hospitalized patients. Consequently, a dedicated information system that integrates nutrition data and decision support into routine workflows is critically needed.

**Objective:**

To construct and evaluate a nutrition management module fully integrated into the Hospital Information System (HIS) to unify information flows and optimize the efficiency of clinical nutrition management.

**Methods:**

Conduct surveys to identify issues within the clinical nutrition management system and analyze clinical needs. Assemble a multidisciplinary expert team to define system functionality and use cases. Translate the functional requirements into code and implement them as a modular application designed for subsequent expansion. Test and debug the system before embedding it into the HIS for deployment. After deployment, evaluate clinical utility through treatment data analysis, and assess software performance and user satisfaction via surveys.

**Results:**

An integrated Clinical Nutrition Management System (CNMS) grounded in an integrative framework was successfully developed. The application adopts a modular design and covers the core steps of the nutritional-care pathway, from nutritional assessment through the prescription of parenteral and enteral nutrition to intake monitoring. Compared to the old system, the new module obtained higher user satisfaction scores (8.48 ± 1.21 vs. 6.70 ± 1.78). Users particularly praised the application’s visualization logic, medication list, real-time alerts, preset PN packages, and knowledge modules.

**Conclusion:**

The application demonstrated acceptable usability and user satisfaction. Its logic-driven, workflow-integrated design pattern, combined with potential for scalability, holds promise for scaled interdisciplinary implementation. This system may support more structured nutrition management and workflow efficiency; however, its impact on clinical outcomes such as complications, length of stay, and mortality was not evaluated in this study and should be examined in future research.

## 1 Introduction

The incidence of malnutrition among hospitalized patients ranges from 20 to 50%. Malnutrition is associated with increased complications, higher mortality rates, and a significant rise in healthcare costs (1, 2). Nutrition as an integral part of treatment protocols, is considered not merely supportive but therapeutic (3, 4). Appropriate nutritional therapy can improve patient outcomes and reduce healthcare costs (5, 6). Compared to other treatments, nutritional therapy involves a more complex process and requires a higher degree of individualization. This process demands real-time integration of laboratory, anthropometric, and clinical data to allow dynamic regimen adjustments (7). However, most physicians have not received specialized training in medical nutrition therapy (8). Consequently, physicians without formal nutrition training frequently prescribe inappropriate or even unsafe nutritional regimens (9, 10).

Overburdened physicians must monitor numerous inpatients daily (11). At the same time, they provide outpatient care, perform surgery, and conduct research (12, 13). In most current hospital information systems (HIS), nutritional data are dispersed across multiple modules, forcing clinicians to switch repeatedly between screens to assess and manage patients’ nutritional status. Such fragmented workflows disrupt clinical routines and create additional, often invisible, repetitive workload (14, 15). Excessive workload elevates the risk of medical errors and compromises patient outcomes (16, 17). Clinical decision-support systems (CDSS) integrate evidence-based knowledge with individual patient data to streamline clinical decision-making, thereby enhancing care quality and patient outcomes (18).

Experts from the American Society for Parenteral and Enteral Nutrition (ASPEN) recommend in their systematic guidelines that electronic health records integrate intake, anthropometric, and biochemical data to provide a comprehensive basis for individualizing nutrition monitoring (19). However, existing nutrition information systems reported to date focus either on parenteral-nutrition dose calculation or on enteral-nutrition monitoring records, lacking a systematic information framework for comprehensive clinical nutrition management (20–23).

We therefore developed an integrated Clinical Nutrition Management System (CNMS). The system integrates multi-source information, such as electronic medical records and laboratory data into a single visual interface with structured information modules, and consolidates the operational menus for nutritional diagnosis and treatment. Medical staff can conveniently obtain patients’ nutritional information and devise individualized nutrition plans without switching repeatedly between screens. The remainder of this paper describes the system design and development, its interface and core functions, and evaluation results.

## 2 Methods

### 2.1 Study design

This is a single-center, uncontrolled pre–post implementation study on the development and application of a clinical information system. The study was conducted at a large public hospital in China and comprised the design and application of the information system, as well as a cross-sectional survey evaluating the system after its implementation.

The study involved five sequential phases: (1) a pre-development needs assessment in 2021; (2) project approval, requirement consolidation, and system design in 2022; (3) system development, testing, and integration into the HIS between 2022 and 2024, including intellectual property registration in 2024; (4) a retrospective quality evaluation study in 2025, involving a pre–post evaluation of prescription standardization and working time; and (5) a cross-sectional user survey conducted in early 2026 to evaluate the usability and user experience of the system.

### 2.2 Clinical investigation and needs identification

In 2021, as part of our routine medical quality improvement initiatives, we conducted an open-ended questionnaire survey via the institution’s internal online platform, focusing on “existing issues and optimization suggestions for parenteral and enteral nutrition medication.” The survey targeted 5,845 physicians, pharmacists, and nurses directly involved in patient nutrition care, yielding 1,090 valid responses (an effective response rate of 18.65%). Qualitative analysis of the feedback revealed that 444 respondents explicitly expressed an urgent need to optimize the existing information system. They identified four core pain points: low differentiation among nutritional medications, a lack of transparency in drug catalogs and inventory information, poor continuity in cross-module workflows, and the cumbersome, time-consuming nature of calculating energy and nutrient dosages. Based on these findings, we distilled the core user requirements into three key areas: constructing a unified platform integrating patient nutrition management functions; providing clinical decision support tools for personalized nutrition planning; and offering specialized medical nutrition information support.

### 2.3 Administrative approval and team formation

In 2022, based on the findings of the above quality management work, we planned to develop a new information system to assist physicians in managing clinical nutrition therapy for inpatients. The identified requirements in the preliminary survey were consolidated into a formal project proposal and submitted to the hospital administration for feasibility review. The project was approved and allocated the necessary budget. To ensure professional development, a multidisciplinary team was established, comprising clinical nutritionists, hospital pharmacists, attending physicians, and senior IT engineers. Each member’s responsibilities and the collaboration workflow were explicitly defined.

### 2.4 Solution design

Based on multiple rounds of prior communication, we convened a formal face-to-face focus-group discussion to finalize the design of the information system. Input was gathered from representatives across IT, clinical medicine, pharmacy, nutrition, and nursing, with final decisions made by the study leads. Disagreements were resolved through consultation with external experts and a show-of-hands voting mechanism. Ultimately, the expert panel distilled the clinical requirements into four core functions: proactive nutrition-information display, real-time therapy monitoring, automated regimen calculation, and an embedded knowledge base. The system was required to be fully integrated into the Hospital Information System (HIS) to enable real-time data exchange, with an interface designed for intuitive operation and ease of use.

### 2.5 Program development

The system development was carried out between 2022 and 2024. The information related to the system development is as follows:

#### 2.5.1 Development strategy

The clinical expert panel and the medical software engineering team collaboratively confirmed the functional requirements outlined in phase (3) and formulated a comprehensive development strategy. The technical team selected Visual Studio 2017 as the development environment and implemented the core modules in C# within the. NET Framework, which is the standard language for enterprise-level HIS integration at our institution. Adopting an agile development approach, they successfully converted the code into an operational program. A modular, plug-and-play architecture was employed to facilitate system operations, enabling independent module updates and dynamic expansion. Following thorough integration testing, the software was successfully incorporated into the institution’s HIS and subsequently deployed for clinical use.

#### 2.5.2 System integration architecture

CNMS retrieves patient demographic, diagnostic, and laboratory data through direct database views and stored procedures that are securely configured within the HIS database. This architecture ensures read-only access to sensitive clinical information, enabling near-real-time synchronization without interfering with transactional operations of the primary HIS. All data exchanges are logged for audit purposes. Upon completion of coding, the software underwent systematic functional testing through predefined test cases to identify and rectify potential software defects.

#### 2.5.3 User interface and workflow

Within the nutritional information display interface, text boxes provide a preview of nutritional data. Buttons initiate operations such as calculations or updates, while dropdown menus offer selectable options to streamline the clinical workflow.

#### 2.5.4 Calculation logic, alert rules, and validation

The therapeutic recommendations and calculation rules in CNMS are based on current treatment guidelines and expert consensus statements. In accordance with authoritative Chinese clinical nutrition guidelines, nutritional targets for general adult patients are set at 25–30 kcal/kg/day for energy and 1.0–1.5 g/kg/day for protein (24).

Enteral nutrition calculations are primarily based on the official product inserts of enteral nutrition formulations. The nutritional parameters of each product, including nutrient content per milliliter or per gram, were entered into the system backend. The system then calculates each patient’s daily nutritional intake by multiplying the nutrient density by the prescribed dose and dosing frequency.

For parenteral nutrition, energy calculations use the following conversion factors: 3.4 kcal/g for glucose, 4 kcal/g for amino acids, and 9.0 kcal/g for fat emulsion (25, 26). When enteral and parenteral nutrition are administered concurrently, the system sums energy, protein, and fluid volume from both sources and displays the combined results on the main interface. Cation concentrations and osmolarity are calculated by converting drug doses to moles based on molecular weight and then determining molar concentrations relative to the total volume.

Safety limit alerts are triggered when calculated values exceed predefined thresholds derived from clinical expert consensus and practice guidelines, including Chinese adaptations of international guidelines. These thresholds cover parameters such as maximum infusion rates, electrolyte limits, and osmolarity (27, 28). Since the osmolarity can be managed by selecting an appropriate venous access route, we have chosen to display the value in red font as a visual reminder for clinicians in the system alert design, rather than triggering an automatic alert. In CNMS, automatic alerts are triggered for four variables—monovalent-cation concentration, divalent-cation concentration, final glucose concentration, and final amino-acid concentration—because out-of-range values may compromise the compatibility and physicochemical stability of the parenteral admixture or precipitate adverse infusion reactions, thereby posing a significant safety risk. The remaining variables displayed in the prescription-parameters panel—total volume, total energy, total amino acids, total nitrogen, glucose energy, lipid energy, glucose-to-lipid energy ratio, energy-to-nitrogen ratio, energy per kilogram of body weight, amino acids per kilogram of body weight, osmolarity, and glucose-to-insulin ratio—are presented as reference ranges to support clinical judgement and do not currently trigger automatic alerts; an out-of-range value of a reference-only variable is displayed in red font only, to prompt physician review.

Prior to clinical implementation, the system’s calculation logic and alert rules were validated using five representative prescription scenarios: a routine adult prescription with all decision variables within their reference ranges; a borderline prescription positioned just within the osmolarity reference range (≤900 mOsm/L); and three deliberately out-of-range prescriptions—one triggering the final amino-acid concentration alert, one with osmolarity exceeding the reference range without triggering an alert, and one triggering multiple alerts simultaneously.

For each scenario, the values generated by the CNMS were compared with values obtained by independent manual calculation, and the alerts generated by the system were compared with the alerts expected from the predefined thresholds. A scenario was considered verified when the alert-triggering variables agreed with the independent calculation and exactly the expected alerts were generated. All five scenarios met these criteria, both before and after formal deployment. The full verification record, including the inputs, the independently calculated values, the CNMS values, the expected and observed alerts, and the pass criteria, is provided as Supplementary 2.

#### 2.5.5 Security, privacy, and governance

CNMS implements role-based access control (RBAC) with three distinct user roles. Physicians have full access to patient data viewing, nutrition risk assessment, and prescription generation; pharmacists have read-only access to prescriptions for review and verification; and administrators are responsible for user account management and system configuration. Access is granted on a strict one-account-per-user basis, and account sharing is prohibited. All user activities are recorded in an immutable audit log for traceability and record review.

CNMS adheres to the institution’s data retention and disaster recovery policies, which include regular off-site backups to ensure data integrity and availability. In the event of system failure or unplanned downtime, predefined emergency procedures are activated to maintain continuity of clinical nutrition care, including fallback to manual prescription workflows.

The system is designed to support, not replace, clinical judgment. Clinicians retain full authority to override any system recommendation when their clinical assessment differs, and all override actions are automatically recorded in the audit trail with timestamps and user identification.

A formal governance process governs all modifications to the system. Changes to clinical rules, backend parameters, and knowledge base content require dual approval from both clinical nutrition experts and the hospital’s information technology department. All changes are subject to version control and maintained in a change log to ensure traceability and accountability.

CNMS has passed the hospital’s internal privacy and cybersecurity review and complies with applicable Chinese regulations and standards.

### 2.6 Application evaluation of CNMS

Following formal integration into the hospital information system (HIS), the project lead delivered user training sessions detailing the system’s functions and operational procedures. Six months after system implementation (in 2025), we conducted a retrospective survey to evaluate the standardization of nutritional prescriptions and the changes in time required for related tasks before and after system adoption (29).

To further evaluate the user experience of the CNMS, we conducted a survey in early 2026 among medical staff from the clinical departments with the highest volume of nutritional therapy at our institution, including digestive surgery, neurology, neurosurgery, and the intensive care unit. The questionnaire (see Supplementary 1) comprised three sections: (i) respondent demographics and professional characteristics; (ii) patterns of CNMS usage (frequency, most-used features); and (iii) usability evaluation. The usability evaluation items employed a 3-point Likert scale (Great/Fair/Poor) to assess interface layout, aesthetics, data presentation, workflow efficiency, and functional adequacy. In addition, based on each respondent’s prior experience with both the new and old systems, a 0–10 numeric rating scale was used to provide a direct comparison between the two systems. The questionnaire was pretested by three healthcare professionals (two physicians and one pharmacist) to assess the clarity and comprehensibility of the items and response options, and the display format was adjusted to improve readability. The eligibility criteria for questionnaire respondents were prespecified before data collection: to be included, respondents were required to use the CNMS routinely in their daily work and to have experience with both the current (new) and the previous system, thereby enabling a meaningful within-respondent comparison of the two systems. These criteria were implemented as automatic skip logic in the online questionnaire. Respondents who reported never having used the nutrition-order functions of either system, having used only one of them, or “almost never” using nutrition-related functions were automatically routed to the end of the questionnaire and did not complete subsequent items. The survey was administered via Wenjuanxing, a widely used Chinese online survey platform, with the link distributed to internal work groups through departmental liaisons. Participation was voluntary, and electronic informed consent was obtained from all participants prior to questionnaire submission. All responses were anonymized.

### 2.7 Statistical analysis

Data analysis was performed as follows. For the questionnaire survey responses, descriptive statistics were used, with frequencies and percentages calculated to summarize the categorical variables. Regarding the comparison of user experience ratings between the new and old systems, no inferential statistical tests were performed given the limited sample size, the subjective nature of satisfaction ratings, and the exploratory aim of observing application trends; results are presented as mean ± SD only.

## 3 Results

A clinical nutrition management system, CNMS, was successfully developed and initially deployed in a single tertiary hospital. The system has been registered for computer software copyright and obtained a design patent certificate by the China National Intellectual Property Administration (*No. 2024SR1242079 and ZL202430428062.7, respectively*). Because the CNMS was designed and deployed in a Chinese-language clinical environment, the figures presented in this manuscript are manually drawn schematic representations of the system interface, with all interface text translated into English for international readability. These schematics are based on actual software screens but were redrawn to facilitate understanding by non-Chinese-speaking readers.

### 3.1 Interface overview of CNMS

The visual integration display module is a key feature of the CNMS. As shown in Figure 1, the module is displayed at the top of the patient-management screen alongside other clinical modules (such as nursing, surgery, and laboratory tests). Clicking the ‘Nutrition Management Module’ tab instantly loads the patient’s nutritional therapy information. Within this interface, users can quickly access key nutritional information, including the patient’s age, gender, height, recent weight, BMI, and nutritional screening score. The patient’s current dietary and nutritional intake status can also be previewed, including EN and PN, medically specialized foods and dietary order types, as well as the total volume, energy, and protein content of these nutritional sources. The interface incorporates a medication monitoring window. This window displays all nutrition-related laboratory test results for the patient, with abnormal values highlighted in a conspicuous color. The top right corner of the screen features menus for nutrition consultations and the issuing of nutrition orders, while the bottom right corner contains a nutrition knowledge window.

**Figure 1:**
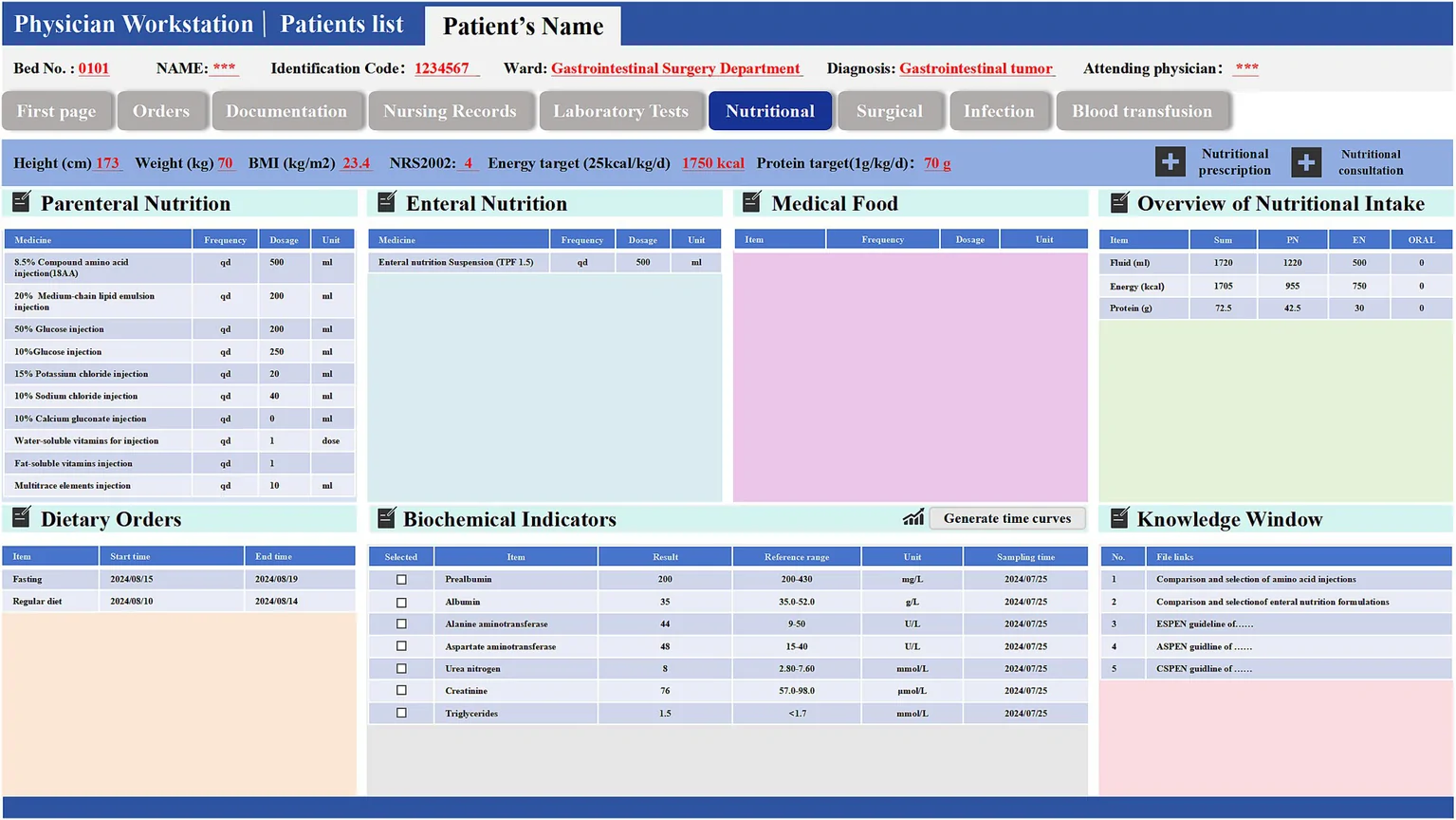
The schematic diagram of the Clinical Nutrition Management System (CNMS). Located at the top of the patient-management screen alongside other clinical modules (e.g., nursing, surgery, and laboratory tests), this module instantly loads nutritional therapy information upon clicking the ‘Nutrition Management Module’ tab. The interface provides quick access to key patient data, including demographics, anthropometrics (height, recent weight, BMI), and nutritional screening scores. It also previews current dietary and nutritional intake (EN, PN, medically specialized foods, and dietary orders), detailing total volume, energy, and protein content. A medication monitoring window displays nutrition-related laboratory results, with abnormal values highlighted. Menus for nutrition consultations and order issuance are located in the top right corner, while a nutrition knowledge window is situated in the bottom right.

### 3.2 Core functions of CNMS

- *Nutritional screening*. If a user prescribes nutritional medication or medical food for a patient without first confirming their nutritional risk or malnutrition diagnosis, the system automatically triggers a nutritional risk screening window. Users must complete the assessment before initiating nutritional therapy, thereby preventing inappropriate treatment.
- *Nutrition consultation*. *U*sers may proactively click the ‘Nutrition Consultation’ tab when necessary to request assistance from a nutrition specialist. If a patient’s nutritional risk screening indicates a higher risk level, the system automatically prompts the nutrition consultation option. Users can then choose whether to accept or decline the recommendation, ensuring that patients receive professional nutritional management.
- *Treatment plan recommendation*. In the process of designing a nutritional plan, users can enter the patient’s nutrient deficiency ratios. The system then recommends appropriate protein and energy regimens, along with corresponding intake levels for the three major nutrients. Users can then utilize these recommendations to select suitable drug dosages and formulate personalized nutritional treatment plans.
- *Nutritional prescription design*. When the user clicks on the “Nutrition Orders” menu option, two selection buttons appear on the left-hand side: “Enteral Nutrition” (EN) and “Parenteral Nutrition” (PN). When the EN medication menu is selected, CNMS displays a list of potential medications (see Figure 2). Users can select the required EN medications and enter the corresponding dosage, frequency, administration rate, and payment type. When the user clicks “Calculate,” the system computes and displays the precise daily values for energy, fluid volume, protein, electrolytes and dietary fiber provided by the order. If multiple medications are selected, the system automatically adds together the nutritional parameters of all the selected items to derive the total nutritional parameters for the order. The EN calculation process is relatively straightforward and is primarily based on the nutritional composition of the selected medications.
  If the user selects PN medications, CNMS offers two options: “Customized” and “Universal.” If “Customized” is selected (as shown in Figure 3), the system displays a list of all available medications categorized by glucose, fat, amino acids, electrolytes, vitamins, and trace elements. Users can then select the required nutrient specifications and dosages for each patient, thereby ensuring that no essential components are omitted. During drug selection, the calculation window on the right displays real-time parameters such as energy, protein, the glucose-to-lipid ratio, thermic nitrogen ratio, and electrolyte concentrations. If any ratio exceeds safe limits, the system highlights the value in red and displays adjustment suggestions when the mouse hovers over it. Users can then modify the regimen based on the prompts until the warning disappears, ultimately generating an appropriate, personalized PN prescription.
  If users do not require personalized prescriptions, they may select the “Universal” option (as shown in Figure 4). The system then displays a list of preset packages named after primary parameters, such as energy, fluid volume, and protein. Users can select their preferred package and click the “Save” button to apply the preset to the patient. They can also adjust medications within the package as needed. The modified settings can be saved as a personal or team preset for quick access in the future, saving time on designing and entering prescriptions. Administrators can enter and modify the calculation formulas, alert rules, and preset packages for these functions in the system backend.
- *Nutritional therapy monitoring.* The main interface allows users to swiftly review a patient’s current dietary regimen, including information on fasting or specialized diabetic diets, and to access real-time intake data for energy, fluid volume, and protein dosage delivered through various nutritional pathways. The laboratory monitoring window below enables users to review recent laboratory test results and select indicators to generate trend lines to determine whether the nutritional plan requires adjustment.
- *Personalized Medical Payment*. Given the distinctive characteristics of PN, which encompasses the administration of multiple medications within a single prescription, CNMS devised payment options (insurance/self-pay category) for each medication, facilitating flexible matching with treatment requirements and health insurance types.
- *Knowledge window*. The CNMS page features a nutrition information panel in the bottom-right corner. Users can access the required nutritional information by clicking the relevant heading. Pharmaceutical information services provide comprehensive data on commonly used nutritional supplements. These data include nutritional components, dosage forms, specifications, special attributes, contraindications, drug prices, and medical insurance payment restrictions. Furthermore, the window provides the latest therapeutic guidelines to help users update their treatment strategies. Content is updated quarterly by a dedicated content committee comprising clinical pharmacists and dietitians, with a formal review and approval workflow to ensure clinical safety. Meanwhile, the window has reserved an interface for AI-assisted nutritional diagnosis and treatment, allowing for further program expansion.
- *Backend parameter management*. *System* and content updates are critical throughout the CDSS life cycle (14). Administrators set parameters in the CNMS backend, which are converted into display values and output to the user interface. When institutional drugs are adjusted, or guidelines are updated, administrators modify backend parameters to ensure users have access to the most recent reference data at the front end.

**Figure 2:**
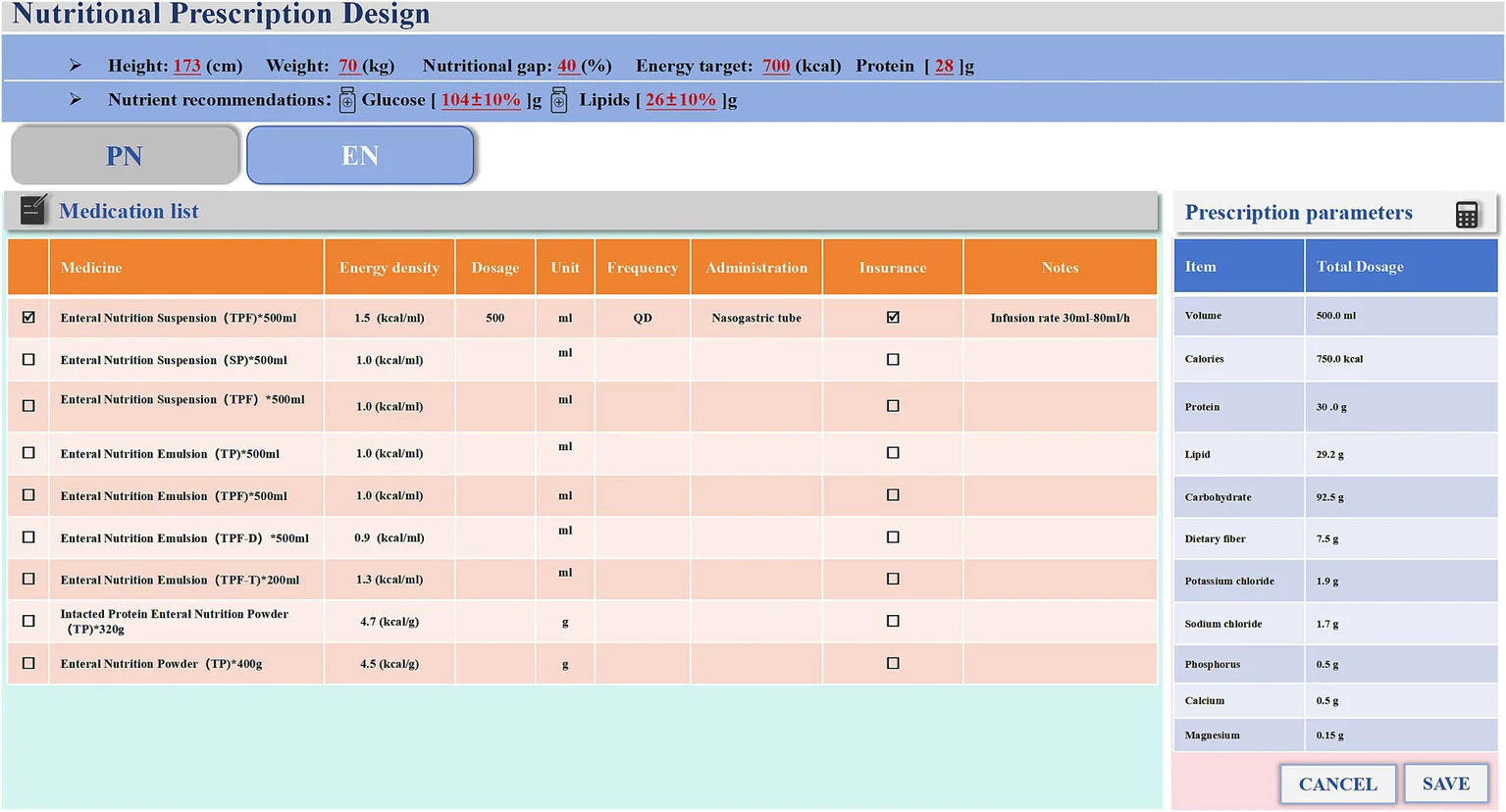
The schematic diagram of the prescription of enteral nutrition. Clinicians select EN medications and input parameters including dosage, frequency, administration rate, and payment type. The system automatically computes the precise daily totals for energy, fluid volume, protein, electrolytes, and dietary fiber. For multiple medications, nutritional parameters are cumulatively summed to derive the total values for the order.

**Figure 3:**
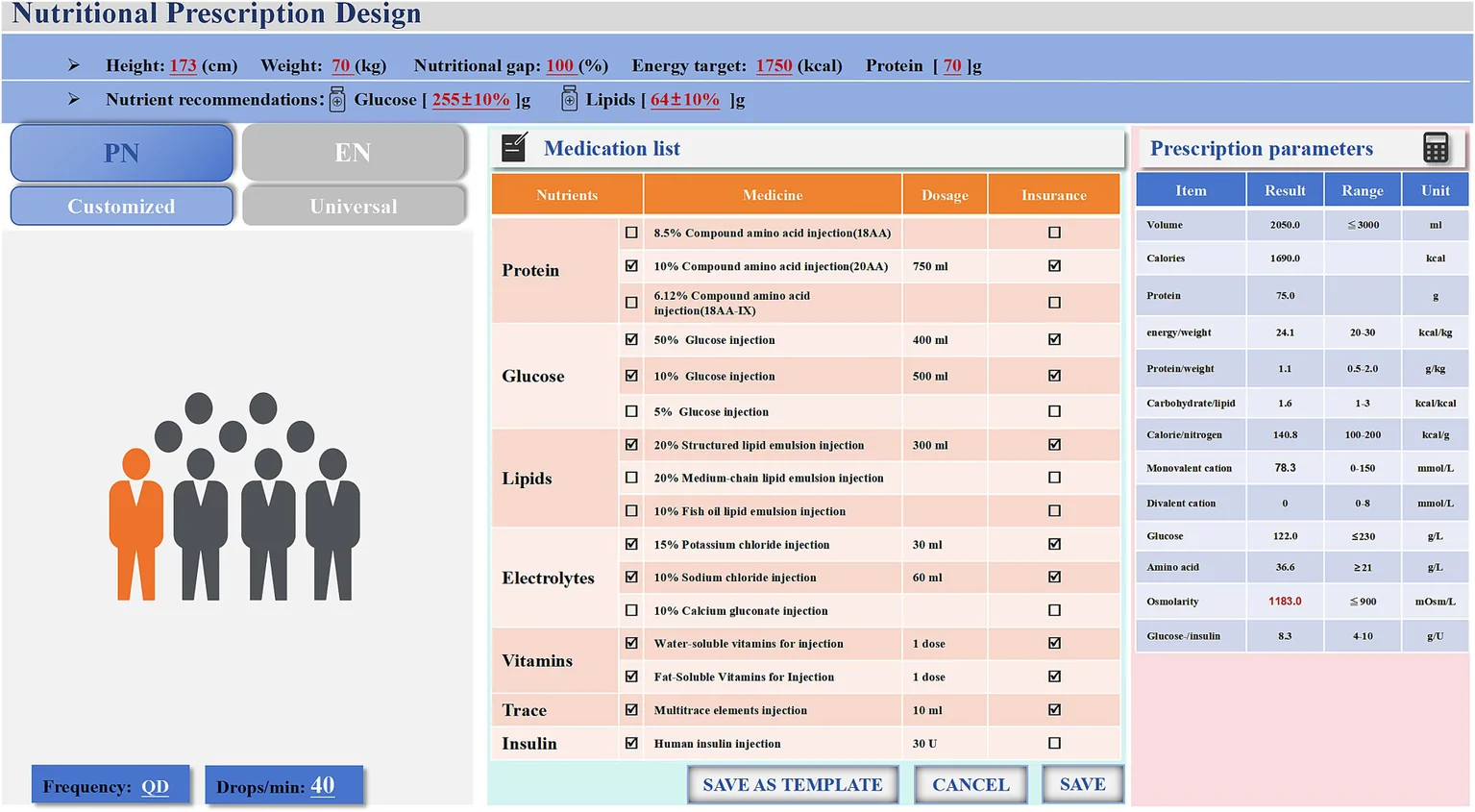
The schematic diagram of customized parenteral nutrition prescription. The system presents categorized nutrient options (glucose, lipids, amino acids, etc.) for clinicians to select specific medications and dosages, while providing real-time calculation of parameters like energy and the glucose-to-lipid ratio. Values exceeding their reference ranges are highlighted in red. Prescriptions in which one of the four alert-triggering variables (monovalent-cation, divalent-cation, final glucose, or final amino-acid concentration) is out of range cannot be saved and must be corrected before proceeding; out-of-range reference-only values, such as osmolarity, are highlighted in red only and do not prevent saving.

**Figure 4:**
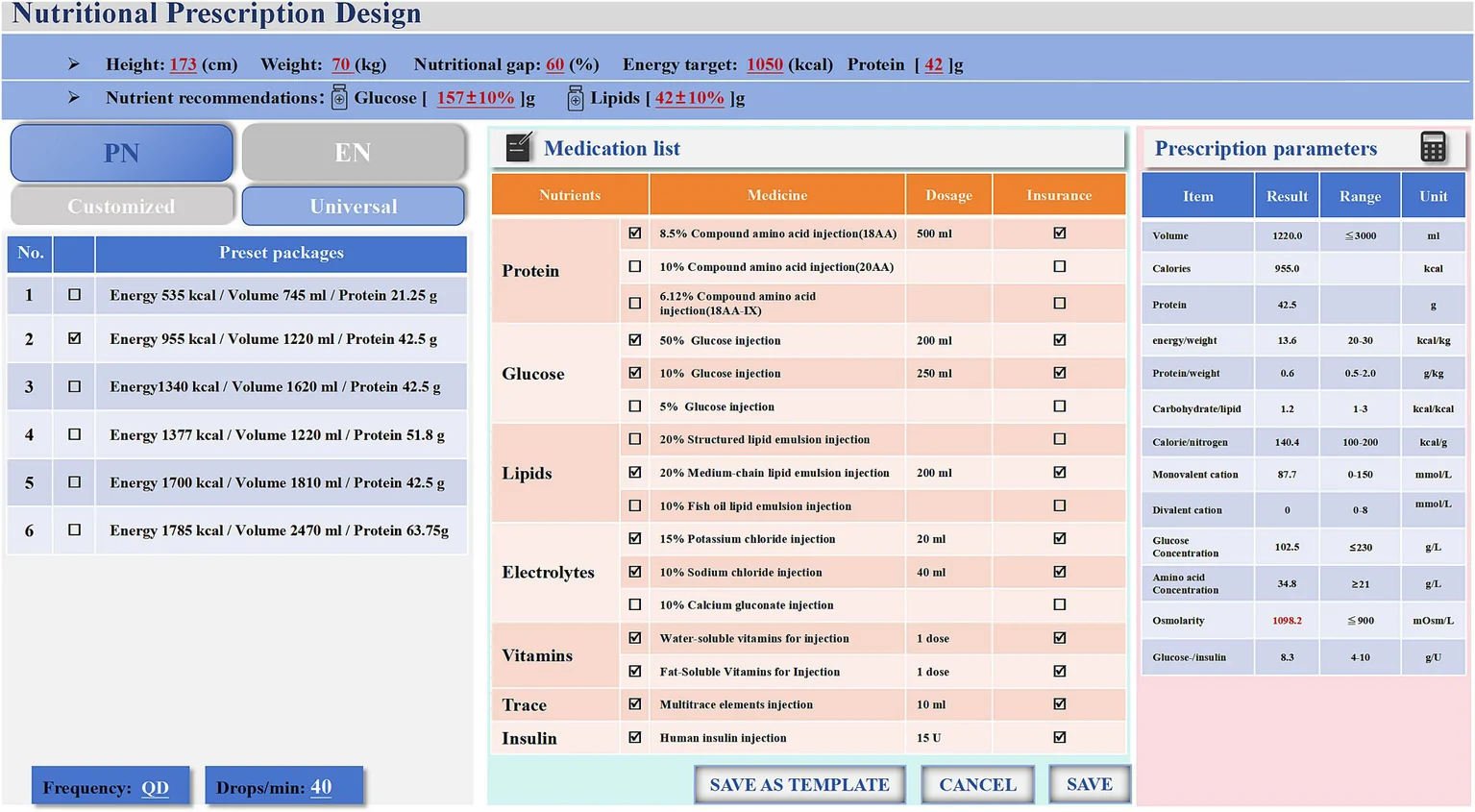
The schematic diagram of universal preset packages of parenteral nutrition. Preset packages based on energy, fluid volume, and protein are available for clinician selection and customization. Adjusted settings can be saved as personal or team presets to optimize workflow. System administrators retain the ability to modify calculation formulas, alert rules, and preset packages in the backend.

### 3.3 CNMS application evaluation

A total of 72 respondents entered the survey. Of these, 24 did not meet the prespecified eligibility criteria and were routed out of the questionnaire by its automatic skip logic: 19 had insufficient experience of the two systems (10 had never used the nutrition-order functions of either system, 6 had used only the previous system, and 3 had used only the current system), and 5 reported that they “almost never” used the nutrition-related functions. The remaining 48 respondents were therefore eligible and were included in the analysis (*Table* *1*). Of the respondents, 54.17% were female and 45.83% were male. 54.17% of respondents were aged 31–40 years. In terms of professional roles, clinicians constituted the majority at 83.33%, followed by dietitians and nurses. Practice areas covered included surgery, internal medicine, and intensive care. Most participants had practiced clinical nutrition for more than 1 year. Regarding software usage frequency, over 70% of respondents used CNMS at least once per week. The most frequently utilized modules out of the 13 available were “Calculation of PN Prescription,” “Presets of PN Prescription,” “Nutrition Prescription Function,” “Calculation of Enteral Nutrition,” and “Nutritional Intake Overview.”

**Table 1:** Results of the questionnaire on software usability.

Question	Results
Personal information of respondents (*n* = 48*)	
What’s your gender?	• Male (22, 45.83%) • Female (26, 54.17%)
What is your age range (y)?	• 25 ~ 30 (12, 25.00%) • 31 ~ 40 (26, 54.17%) • 41 ~ 50 (9, 18.75%) • > 50 (1, 2.08%)
What’s your identity?	• Physician (40, 83.33%) • Nutritionist (6, 12.50%) • Nurse (2, 4.17%)
What is your major?	• Surgery (21, 43.75%) • Internal Medicine (13, 27.08%) • Critical Care Medicine (12, 25.00%) • Others (2, 4.17%)
How long have you been involved in clinical nutrition?	• 1 ~ 3 y (20, 41.67%) • 4 ~ 6 y (11, 22.92%) • ≧7 y (17, 35.41%)
Applications of CNMS	
What is the frequency of CNMS usage?	• Every month (14, 29.17%) • Every week (24, 50.00%) • Every day (10, 20.83%)
What are the top 5 most frequently used features in CNMS?	• Calculation of PN Prescription (30) • Presets of PN Prescription (25) • Nutrition Prescription Function (23) • Calculation of Enteral Nutrition (20) • Nutritional Intake Overview (16)
Evaluation of CNMS	
Do you think the interface layout is reasonable?	• Great (37, 77.08%) • Fair (10, 20.83%) • Poor (1, 2.08%)
How would you evaluate the appearance and aesthetics of the CNMS?	• Great (27, 56.25%) • Fair (20, 41.67%) • Poor (1, 2.08%)
Does the presentation of patient data feel user-friendly and straightforward?	• Great (30, 62.50%) • Fair (17, 35.42%) • Poor (1, 2.08%)
Does the nutritional prescription-writing process simplify and streamline workflow?	• Great (21, 43.75%) • Fair (22, 45.83%) • Poor (5, 10.42%)
Do the presets for PN meet your work requirements?	• Great (28, 58.33%) • Fair (17, 35.42%) • Poor (3, 6.25%)
Compared to input-based medication menus, what advantages do list-based medication menus offer? (Check all that apply)	• The speed of prescription calculation and entry has improved (38, 79.17%) • A wider range of medication options is available (32, 66.67%) • Caused inconvenience (5, 10.42%) • No advantages (2, 4.17%)
Is CNMS’s current functionality sufficient?	• Great (30, 62.50%) • Fair (16, 33.33%) • Poor (2, 4.17%)
How would you assess the functionality of the Nutrition Knowledge window?	• Great (34, 70.83%) • Fair (13, 27.08%) • Poor (1, 2.08%)
Please rate the new and old systems (0 ~ 10)	• New (8.48 ± 1.21) • Old (6.70 ± 1.78)
How likely are you to recommend this software to your colleagues?	• Highly (44, 91.67%) • Generally (4, 8.33%) • Others* (0, 0)

^*^Of the 72 respondents who entered the survey, 24 were routed out by the prespecified eligibility criteria (Section 2.6) and 48 were eligible; all percentages in this table are based on these 48 respondents. “Others” was a predefined response option representing non-recommendation or abstention; no participants selected this category.

Users gave CNMS an overall positive evaluation. Over 77% of respondents found the interface layout well organized, 56.25% were satisfied with its visual appeal, and 62.50% considered the presentation of patient data intuitive and user-friendly. In terms of functionality, 58.33% of respondents found the preset PN packages adequate for their needs, while 43.75% believed the prescription workflow streamlined their tasks. The list-based medication menu was considered to have clear advantages in accelerating prescription entry (79.17%) and offering expanded drug options (66.67%). Additionally, 70.83% of users rated the nutrition knowledge window positively, and 62.50% considered the current functionality of the system to be relatively complete. A comparison of scores between the new and old systems showed higher ratings for the new system (8.48 ± 1.21 vs. 6.70 ± 1.78). Ultimately, 91.67% of respondents expressed a willingness to recommend the system to their colleagues.

## 4 Discussion

CNMS is an innovative system for managing nutrition information. Numerous reports have described information systems that assist clinical nutrition therapy. For example, computer systems that calculate prescriptions for PN can streamline workflow for pharmacists and doctors and help prevent errors during medicine preparation (20–22, 30). In one instance, pharmacists had developed a computer program that calculates nutrition. The program can evaluate, assess, and calculate personalized prescriptions for patients and improve the efficiency and effectiveness of nutritional support therapy. However, this system is limited to multidisciplinary team treatment in radiation oncology and intensive care units (31). Moreover, its hardware interface relies on a mobile device requiring manual entry of patient parameters and is not fully integrated with the HIS, which limits its broader clinical applicability. Pedro and his team described an ideal software design for managing nutrition (32). The software could automatically screen, monitor, assess patients’ nutrition, and create personalized nutrition care plans; it also could provide prescription assistance. However, the subsequent development progress and application outcomes of this software have not been reported. In contrast, our CNMS is distinguished by its deep integration into the HIS through a database-level architecture that enables near-real-time data retrieval without disrupting existing clinical workflows. This design choice was made possible by institutional administrative support, which mitigated common barriers such as data security concerns and interface complexity. The integrated approach not only reduces development overhead but also eliminates the need for users to manually enter or reconcile data across separate systems.

Research indicates the potential advantages of integrated, personalized nutrition management systems in enhancing nutrition support processes and care quality, particularly in improving data accuracy and operational efficiency (33). The Roadmap for National Clinical Decision Support published by the American Medical Informatics Association (AMIA) identified three pillars for effective CDSS: knowledge availability when required, high levels of adoption, and effective utilization. Furthermore, there is a necessity for continuous enhancement of Computerized Decision Support (CDS) (34). Depending on the level of integration with the HIS, CDS can operate as a standalone system or be integrated into clinical systems. It is further noted that more advanced systems may provide pathway-based services or treatment decision support models (35). It is recommended by ASPEN experts that information systems integrate intake data, anthropometric measurements, and biochemical data to facilitate personalized adjustments to clinical nutrition monitoring protocols (19).

The system design explicitly addresses these recommendations by providing a unified dashboard that aggregates anthropometric, laboratory, and intake data, and by embedding decision support directly into the prescribing workflow. The two-option approach for PN prescriptions (Customized vs. Universal) reflects a pragmatic balance between flexibility for complex cases and efficiency for routine scenarios—a design choice that emerged from our initial needs assessment. The CNMS is fully integrated with the HIS of the host medical institution. This integration facilitates the further consolidation of clinical data. In practice, CNMS enables users to calculate nutritional prescription parameters and provides treatment goal recommendations. These features assist users in comprehensively and efficiently managing patient nutritional therapy.

The CNMS enhances user satisfaction and productivity. The evaluation of decision-support systems remains challenging. As early as 2003, Berner proposed that an ideal decision support system should possess the following elements: producing the correct diagnosis, offering appropriate management recommendations, ensuring user satisfaction, and optimizing operational experience to deliver comprehensive support (36). We referenced an evaluation method for a system that diagnoses and treats rare diseases (37). A user satisfaction survey questionnaire was developed, with a focus on user evaluations of the CNMS interface, usability, functional accessibility, and overall satisfaction ratings. Users were invited to assess performance using simulated cases. We acknowledge that our evaluation relies on an internally developed questionnaire without formal psychometric validation, and the respondent sample predominantly comprised physicians (83.3%) with limited nursing input. Future evaluations should include more balanced multidisciplinary representation and validated instruments.

The nutrition module was integrated into the patient management interface of the hospital HIS as a series of cards, thereby enabling users to swiftly access patient nutritional information. The utilization of structured, color-coded modules facilitates the categorization of functions, with high-frequency areas placed in a prominent position and low-frequency areas at the periphery. Fixed information is displayed at the top, while dynamic information modules occupy the visual center, helping users quickly grasp key details. Survey results indicate that the interface design received high praise from users.

In the nutritional prescription module, a list-based medication inventory with dosage and frequency allows users to quickly identify target medications and dosages. This feature synchronizes medication availability data with the institution’s real-time inventory, preventing delays caused by drug formulary changes or stock shortages. The calculation functionality that is most critical to users is offered in the form of automated computations, delivering real-time nutritional parameters for prescriptions. For the purpose of nutritional monitoring, key indicators are filtered from extensive databases, including albumin, prealbumin, lipids, blood glucose, and liver/kidney function, and integrated into a dedicated monitoring window. This effectively reduces the time spent retrieving data across modules.

Excessive workloads have been shown to impact the efficiency and quality of medical care. Previous clinical information systems required clinicians to navigate across multiple sections of medical records to assess and monitor inpatients’ nutritional status. This fragmentation added to this burden (15). Such non-clinical task load matters because excessive workload is associated with reduced care efficiency and quality, and with physician burnout. A study of Chinese physicians revealed a correlation between high workload and burnout (38). By consolidating these data into a single dashboard, the CNMS is designed to reduce this non-clinical, information-retrieval burden.

The questionnaire survey results showed that more than 70% of users gave positive feedback on the “Nutrition Knowledge window,” 91.67% of users were willing to recommend this software to colleagues, and the new system’s overall ratings were higher than those for the old system (8.48 ± 1.21 vs. 6.70 ± 1.78), which indicates favourable but preliminary user satisfaction with CNMS. As background, it should be noted that the significant reductions in working time for both physicians and pharmacists during the PN prescription process were reported in our previous study (29). The CNMS is designed to support this workflow by automating calculations and reducing cross-module data retrieval; however, the magnitude of its specific contribution to these reductions remains undetermined.

Pre-development requirements assessment and administrative support are crucial. The design of information products should be user-driven, based on a comprehensive understanding of the needs and usage scenarios of the target audience, to address their concerns and expectations. In the course of developing the CNMS, we used a combination of questionnaires and face-to-face interviews to gather specific requirements and suggestions from medical staff across different departments regarding the nutritional support system. These insights proved pivotal to the system design. This user-centered design philosophy enhanced the system’s practicality and increased user acceptance and satisfaction.

Moreover, the provision of administrative support is equally indispensable throughout the system development and implementation process. The level of administrative backing exerts an indirect influence on the extent to which information systems are updated. In the context of implementing digital systems to support nutrition management, most institutions typically encounter two options. One of these approaches involves the retrofitting of existing clinical information systems, while the other entails the development of a dedicated application that accesses information in target databases through limited interfaces. The former approach generally necessitates the stabilization of additional healthcare resources, the implementation of updates, and upgrades to the institution’s information systems in their entirety. However, it offers superior practical convenience in terms of implementation. The latter generally requires only the design of a standalone system or the use of simple spreadsheets and formulas, with functionality achieved through information transfer between different systems. Nevertheless, this approach is frequently confronted with challenges, including cumbersome usage and the presence of information silos. A considerable number of CDSS function as standalone entities, which hinders their capacity to effectively communicate with other systems. This predicament stems from the inherent difficulty of managing diverse clinical data sources, as well as the complexity of programming. Healthcare institutions frequently exhibit resistance or latent risk perceptions regarding the transmission of sensitive patient information (14, 39). Fortunately, our institution provided robust support for CNMS development, facilitating its integration into the HIS. Consequently, this has led to a reduction in development costs, an enhancement of data security, and a simplification of user workflows.

Several limitations should be acknowledged. First, regarding study design, CNMS was implemented and evaluated at a single tertiary hospital using an uncontrolled, cross-sectional user survey. Ratings of the previous system were obtained retrospectively rather than through contemporaneous measurement and may therefore be subject to recall bias; caution is warranted when generalizing these findings to settings with different patient populations, workflows, or resource levels. Second, regarding measurement, user experience was assessed with an internally developed questionnaire that has not undergone formal psychometric validation. Usability was captured with a 3-point scale only, without standardized instruments such as the System Usability Scale (SUS). A 0–10 numeric rating scale was included for direct comparison between systems, but this single-item score is not equivalent to the SUS, and the two should not be used interchangeably. Third, regarding sample composition, the final analysis included only 48 responses, with physicians comprising 83.3%, nurses only two, and dietitians underrepresented. This distribution reflects the intended user profile of CNMS, which was developed for clinicians with nutrition decision-making responsibilities; in our institution, nurses mainly work within a dedicated nursing information system and access CNMS only occasionally to review patients’ overall nutritional status. The survey therefore targeted these primary users, while retaining nursing responses as a supplementary reference (*n* = 2). Satisfaction findings may therefore not extend to nursing and other professional groups. Moreover, because eligibility was restricted to respondents who used the CNMS routinely and who had experience of both systems, the survey describes this specific group of routine, dual-system users and may not be generalizable to occasional users or to staff without experience of the previous system. Fourth, regarding outcomes, we did not evaluate patient-level clinical outcomes (length of stay, complication rates, mortality) nor process measures such as workload or burnout; the effect of CNMS on clinical outcomes and on users’ work burden remains to be determined. In addition, because the survey was distributed through internal work-group channels, the number of individuals who viewed the invitation is unknown and a precise response rate cannot be calculated. Future multicenter, prospective studies using validated usability instruments, balanced multidisciplinary samples, and patient clinical outcomes are needed to further establish the effectiveness, generalizability, and scalability of CNMS.

## 5 Conclusion

The nutrition management module developed in this study offers a degree of innovation and practical value. It was designed around the comprehensive management of patient nutrition and embeds the relevant workflow into the HIS from an integrative perspective. The impact of this initiative is multifaceted, encompassing the status of nutritional therapy within the HIS, the standardization of the utilization of nutritional medications, and the enhancement of the efficiency of nutritional diagnosis and treatment. These gains, however, were achieved within a combined intervention that also included a parallel pharmacist-led workflow optimization program, and the independent contribution of CNMS cannot be isolated from this combined effect. The initiative has been met with general approval by its users. The design rationale of the CNMS is aligned with current development trends in clinical nutrition, while also providing valuable insights and references for the development of other clinical diagnostic and treatment information systems. Future multicenter clinical studies are still needed to comprehensively evaluate the impact of this informatics solution on patient clinical outcomes.

## Data Availability

The original contributions presented in the study are included in the article/Supplementary material, further inquiries can be directed to the corresponding authors.
