# Supplementary material for: Development and evaluation of an integrated clinical nutrition management system for inpatients
Source: Front Nutr. 2026 Oct 27;13:1971974. doi: 10.3389/fnut.2026.1971974 (PMC13748280; doi:10.3389/fnut.2026.1971974)
Supplement: Supplementary file 1 [file Table_1.DOCX]

问卷调查表

**Questionnaire Survey**(https://www.wjx.cn/)

| **编号No.** | **问题Question** | **答案或备选项AnswerOptions** |
| --- | --- | --- |
| 1 | 请选择您的身份 Please select your role | 本院医生/进修医生/学生/其他 Physician of this hospital / Trainee physician / Student / Other |
| 2 | 请选择您的性别 Please select your gender | 男/女 Male / Female |
| 3 | 请选择您的年龄 Please select your age | 25 ~ 30岁 /31 ~ 40 岁/41 ~ 50岁/>50 岁 25-30 years / 31-40 years / 41-50 years / >50 years |
| 4 | 请选择您的职业 Please select your occupation | 医生/营养师/护士 Physician / Dietitian / Nurse |
| 5 | 请选择您的专业 Please select your specialty | 内科医生/外科医生/重症医生/其他 Internist / Surgeon / Intensivist / Other |
| 6 | 请选择您的职称 Please select your professional title | 初级/中级/高级 Junior / Intermediate / Senior |
| 7 | 请选择您的工作年限 Please select your years of work experience | 1-3年/4-6年/>6年 1-3 years / 4-6 years / >6 years |
| 8 | 请选择您接触临床营养的时间 Please select the length of time you have been involved in clinical nutrition | 1~3年/4~6年/≧7年 1-3 years / 4-6 years / >=7 years |
| 9 | 您是否接受过该软件操作相关的培训？ Have you received any training related to the operation of this software? | 是/否 Yes / No |
| 10 | 新/旧版HIS系统营养医嘱功能使用情况 Your experience with the nutrition order functions of the new/old HIS systems | 从未使用/仅使用过旧系统/仅使用过新系统/使用过新旧系统（黄色填充项为跳转排除调查对象） Never used / Used only the old system / Used only the new system / Used both the new and old systems (items highlighted in yellow indicate respondents excluded by skip logic) |
| 11 | 营养模块相关功能使用频率 Frequency of use of the nutrition module functions | 每天/每周/每月/几乎不用（黄色填充项为跳转排除调查对象） Daily / Weekly / Monthly / Rarely used (items highlighted in yellow indicate respondents excluded by skip logic) |
| 12 | 我院营养管理模块的功能有哪些？（多选） Which functions does the nutrition management module of our hospital provide? (multiple choice) | 肠外营养处方参数计算/肠外营养协定处方模版/肠内营养药品含量和成分计算/患者营养摄入(能量、蛋白、液量)概况预览/患者体重、身高、BMI预览/患者营养缺口录入和营养方案推荐/肠外和肠内营养医嘱开立/营养生化指标预览/营养会诊申请/营养风险筛查/营养知识窗口/饮食医嘱预览/特医食品预览 Parenteral nutrition prescription parameter calculation / Standardized (protocol) parenteral nutrition prescription templates / Calculation of nutrient content and composition of enteral nutrition products / Overview of patient nutritional intake (energy, protein, fluid volume) / Preview of patient weight, height and BMI / Entry of patient nutritional deficits and recommended nutrition plans / Order entry for parenteral and enteral nutrition / Preview of nutritional biochemical indicators / Nutrition consultation requests / Nutritional risk screening / Nutrition knowledge portal / Preview of diet orders / Preview of foods for special medical purposes |
| 13 | 营养模块中您使用频率最高的功能为:（多选） Which functions of the nutrition module do you use most frequently? (multiple choice) | 肠外营养处方参数计算/肠外营养协定处方模版/肠内营养药品含量和成分计算/患者营养摄入(能量、蛋白、液量)概况预览/患者体重、身高、BMI预览/患者营养缺口录入和营养方案推荐/肠外和肠内营养医嘱开立/营养生化指标预览/营养会诊申请/营养风险筛查/营养知识窗口/饮食医嘱预览/特医食品预览 Parenteral nutrition prescription parameter calculation / Standardized (protocol) parenteral nutrition prescription templates / Calculation of nutrient content and composition of enteral nutrition products / Overview of patient nutritional intake (energy, protein, fluid volume) / Preview of patient weight, height and BMI / Entry of patient nutritional deficits and recommended nutrition plans / Order entry for parenteral and enteral nutrition / Preview of nutritional biochemical indicators / Nutrition consultation requests / Nutritional risk screening / Nutrition knowledge portal / Preview of diet orders / Preview of foods for special medical purposes |
| 14 | 界面布局是否合理？ Is the interface layout reasonable? | 合理/一般/不合理 Reasonable / Acceptable / Unreasonable |
| 15 | 系统界面外观（颜色和字体）的美观和视觉舒适程度 The aesthetic appeal and visual comfort of the system interface (colors and fonts) | 很好/一般/很差 Very good / Acceptable / Very poor |
| 16 | 患者数据展示是否清晰？ Is the display of patient data clear? | 清晰/一般/不清晰 Clear / Acceptable / Unclear |
| 17 | 处方开立操作是否简单便捷？ Is the prescription ordering process simple and convenient? | 很好/一般/很差 Very good / Acceptable / Very poor |
| 18 | 肠外营养“协定处方模板”的种类和数量是否能满足您的工作需求？ Do the variety and number of parenteral nutrition "standardized prescription templates" meet your work needs? | 很好/一般/很差 Very good / Acceptable / Very poor |
| 19 | 与旧系统药品录入模式相比，列表式药品菜单有什么特点？ Compared with the medication entry mode of the old system, what are the features of the list-style medication menu? | 提高了处方计算和录入速度/提供了更多可选择的药品/没有优势，且造成了困扰 It improves the speed of prescription calculation and entry / It provides more selectable medications / It offers no advantage and causes confusion |
| 20 | 营养管理模块当前的功能是否完善？ Are the current functions of the nutrition management module adequate? | 很好/一般/很差 Very good / Acceptable / Very poor |
| 21 | 您如何评价营养知识窗口功能？ How do you evaluate the nutrition knowledge portal function? | 很好/一般/很差 Very good / Acceptable / Very poor |
| 22 | 旧系统营养相关操作和功能整体评分(满分10分） Overall rating of the nutrition-related operations and functions of the old system (out of 10) | 0-10分 0-10 points |
| 23 | 新系统营养相关操作和功能整体评分(满分10分） Overall rating of the nutrition-related operations and functions of the new system (out of 10) | 0-10分 0-10 points |
| 24 | 您向同事推荐该软件的可能性？（0-100%） How likely are you to recommend this software to a colleague? (0-100%) | 高度推荐/一般推荐/不推荐或其他 Highly recommend / Moderately recommend / Do not recommend or other |
| 25 | 开放式建议：请提出您对该软件的优化的宝贵建议，我们会认真考虑您的留言并持续改进。欢迎提出开放式建议，比如：您觉得系统存在什么具体问题？您希望增加哪些功能或者元素？您对界面设计有什么建议？您对操作流程和专业性有什么建议？(可不限于上述内容） Open-ended suggestions: Please give your valuable suggestions for optimizing this software; we will carefully consider your comments and continue to improve. You are welcome to provide open-ended suggestions, for example: What specific problems do you think exist in the system? What functions or elements would you like to see added? What suggestions do you have for the interface design? What suggestions do you have for the workflow and professionalism? (Not limited to the above.) |  |
| 26 | 临床研究参与者知情同意（在线签署） Informed Consent for Clinical Research Participants (signed online) | 尊敬的被调查者： Dear respondent: 我们邀请您参加一项“ 临床营养管理系统的开发和应用评价”的临床研究，在您决定 是否参加这项研究之前，请仔细阅读以下内容，它可以帮助您了解该项研究以及为什么要进行这项研究，研究的程序和期限，参加研究后可能给您带来的益处、风险和不便。 We invite you to participate in a clinical study entitled "Development and Application Evaluation of a Clinical Nutrition Management System". Before you decide whether to take part in this study, please read the following information carefully. It will help you understand the study, why it is being conducted, its procedures and duration, and the benefits, risks and inconveniences that participation may bring. 以下是本项研究的介绍： The following is an introduction to this study: 一、研究背景和研究目的 I. Research Background and Objectives 本项目在本机构医疗信息系统中嵌入了一个营养管理模块，在应用过程中，需要收集用户的 体验，进一步优化和完善系统，以提供给用户更好的软件操作性能。 In this project, a nutrition management module has been embedded in the medical information system of this institution. During its use, user experience needs to be collected to further optimize and refine the system so as to provide users with better software performance. 二、具体程序和流程 II. Specific Procedures and Process 您使用本模块各项功能对患者进行营养治疗管理，可被纳入本研究的被调查用户，参与本研 究的在线问卷调查。 If you use the various functions of this module to manage patients' nutritional therapy, you may be included as a surveyed user in this study and participate in its online questionnaire survey. 三、如果参加研究您需要做什么 III. What You Need to Do If You Participate 您需要根据使用体验，对该系统进行评价。 You need to evaluate the system based on your experience of using it. 四、参加本研究可能给您带来的受益 IV. Possible Benefits of Participating in This Study 您作为软件用户，没有直接的获益，但在该软件的后续优化中，为您带来更好的使用体验。 As a software user, you will not receive direct benefits, but the subsequent optimization of the software will provide you with a better user experience. 五、参加本研究可能发生的不良反应、风险以及风险防范措施 V. Possible Adverse Reactions, Risks and Risk Prevention Measures 本研究为非干预性研究，不会增加研究对象额外的相关操作和检查，一般没有不良反应、风 险。 This is a non-interventional study. It will not add any extra related procedures or examinations for the research subjects, and generally there are no adverse reactions or risks. 六、费用情况说明 VI. Statement of Costs 本研究无费用产生。 This study incurs no costs. 七、参与研究的补偿，包括损伤的赔偿 VII. Compensation for Participation, Including Compensation for Injury 本研究为非干预性研究，不会增加研究对象额外的费用，所以没有补偿和相关的赔偿。 This is a non-interventional study and will not incur additional costs for the research subjects; therefore, there is no compensation or related indemnity. 八、您个人信息的保密 VIII. Confidentiality of Your Personal Information 您的医疗记录(包括研究病历及理化检查报告等)将按规定保存在医院。除研究者、伦理 委员会、监察、稽查、药政管理部门等相关人员将被允许查阅您的医疗记录外，其他与研究无关 的人员在未得到允许的情况下，无权查阅您的医疗记录。本研究结果的公开报告将不会披露您的 个人身份。我们将在允许的范围内，尽一切努力保护您个人医疗资料的隐私。 Your medical records (including study case records and laboratory and examination reports, etc.) will be kept in the hospital in accordance with regulations. Except for relevant personnel such as investigators, the ethics committee, monitors, auditors and drug regulatory authorities, who will be permitted to review your medical records, other personnel unrelated to the study have no right to review your medical records without permission. Public reports of the results of this study will not disclose your personal identity. We will make every effort, within the permitted scope, to protect the privacy of your personal medical information. 九、伦理委员会 IX. Ethics Committee 本研究已向浙江大学医学院附属第二医院人体研究伦理委员会报告，经委员会的全面审查包括对受试者的风险评估，并获得了批准。 This study has been reported to the Human Research Ethics Committee of the Second Affiliated Hospital, Zhejiang University School of Medicine. Following the committee's comprehensive review, including assessment of the risks to subjects, approval has been obtained. 提交本问卷即被认为签署研究参与者知情同意 Submitting this questionnaire is deemed as signing the informed consent for research participants. 请您客观回答问卷，这将有助于我们了解该软件的现实可用性，并可为我们进一步改进软件提供参考 Please answer the questionnaire objectively. This will help us understand the real-world usability of the software and provide a reference for further improving it. |
